# Temporal Hierarchy of Hydrogels and Orthobiologic Therapies for Knee Osteoarthritis

**DOI:** 10.3390/gels12070608

**Published:** 2026-07-08

**Authors:** Fábio Ramos Costa, Rubens Martins, João Protásio Netto, Vinicius Calumby Costa, Gabriel Azzini, André Kruel, Tomas Mosaner, Lucas Furtado da Fonseca, José Fábio Lana

**Affiliations:** 1 Department of Orthopedics, FC Sports Traumatology, Salvador 40296-210, BA, Brazil; viniciuscalumby@gmail.com; 2 Medical School, Tiradentes University Center, Maceió 57038-000, AL, Brazil; rubensdeandrade@hotmail.com; 3 Department of Orthopedics and Traumatology, Federal University of Tocantins (UFT), Palmas 77001-090, TO, Brazil; 4 Regenerative Medicine, Orthoregen International Course, Indaiatuba 13334-170, SP, Brazil; drgabriel.azzini@gmail.com (G.A.); kruel.andre@gmail.com (A.K.); tmosaner@uol.com.br (T.M.); ffonsecalu@gmail.com (L.F.d.F.); josefabiolana@gmail.com (J.F.L.); 5 Department of Orthopedics, Brazilian Institute of Regenerative Medicine (BIRM), Indaiatuba 13334-170, SP, Brazil; 6 Medical School, Max Planck University Center (UniMAX), Indaiatuba 13343-060, SP, Brazil; 7 Clinical Research, Anna Vitória Lana Institute (IAVL), Indaiatuba 13334-170, SP, Brazil; 8 Medical School, Jaguariúna University Center (UniFAJ), Jaguariúna 13911-094, SP, Brazil

**Keywords:** knee osteoarthritis, hydrogels, hyaluronic acid, orthobiologics, viscosupplementation, therapeutic persistence

## Abstract

Knee osteoarthritis is commonly managed with intra-articular therapies that differ widely in composition, mechanism, and clinical persistence, yet the duration of their benefit is discussed inconsistently, which limits practical comparison between ozone, hyaluronic acid, platelet-derived products, and cell-rich orthobiologics. In this narrative review we examine the mechanism-driven temporal behavior of these therapies, focusing on the physicochemical, biomechanical, biological, and regenerative factors that influence how long a clinical response persists. By temporal hierarchy we mean the mechanistically informed pattern by which therapies differ in the duration of their physicochemical presence, biological activity, and clinical benefit, not a ranking of efficacy or a claim of equivalence. Read against this definition, ozone occupies the shortest end of the spectrum; conventional hyaluronic acid shows intermediate persistence; modified hyaluronic acid hydrogels may extend activity through improved rheology and enzymatic resistance; platelet-rich plasma and injectable platelet-rich fibrin provide more sustained biological signaling; and bone marrow aspirate concentrate and stromal vascular fraction may act over longer periods through trophic and paracrine pathways. Heterogeneity in study design, patient selection, and outcome reporting still limits firm conclusions, and standardized reporting of product characteristics and time-related endpoints will be essential to validate or refine the proposed framework.

## 1. Introduction

Knee osteoarthritis (KOA) is one of the leading causes of pain, functional limitation, and disability worldwide, and it places a heavy burden on patients and health systems [1]. Cartilage degeneration, synovial inflammation, subchondral bone remodeling, and altered joint mechanics together produce a chronic, multifactorial disease that usually needs long-term symptomatic management [2,3]. Arthroplasty is effective in advanced disease [4], but there is growing interest in less invasive options that relieve pain, improve function, and postpone surgery [5].

Intra-articular therapies have become central to that effort. Ozone, conventional hyaluronic acid (HA), platelet-rich plasma (PRP), injectable platelet-rich fibrin (iPRF), bone marrow aspirate concentrate (BMAC), and stromal vascular fraction (SVF) are all in routine use, even though protocols, indications, and the strength of the evidence vary widely between them [6,7,8,9]. Most studies ask whether these treatments relieve symptoms and improve function. Far fewer ask a question that matters just as much in the clinic: how long the benefit lasts.

In practice, these therapies behave differently over time. Some work quickly but fade; others take longer to help but hold their effect for months. They are hard to compare directly, because studies differ in design, patient selection, preparation methods, injection protocols, follow-up, and outcome measures [10,11,12]. As a result, how long orthobiologic injections keep working is still poorly defined.

Progress in biomaterials and regenerative medicine has added to this picture. For HA, interest has moved toward molecular structure, rheology, viscoelastic recovery, tribology, and how long the material stays in the joint. Modified HA hydrogels built from amphiphilic domains and dynamic physical networks behave differently from conventional viscosupplements and may interact with tissue and persist longer [13,14,15]. Platelet- and cell-based products add mechanisms of their own, such as growth factor release, modulation of inflammation, interaction with the extracellular matrix (ECM), and trophic signaling, which could prolong biological activity inside the joint [8,16,17,18,19].

This suggests that intra-articular therapies are better understood as mechanistically different interventions with different time courses, not as a single uniform category. That distinction matters for choosing a treatment, setting patient expectations, deciding when to reinject, and developing new products. Our aim in this narrative review is to examine how long the effects of the currently available intra-articular therapies for KOA tend to last, and the physicochemical, biomechanical, biological, and regenerative factors that drive that duration. When we speak of a temporal hierarchy, we mean simply that these therapies can be ordered by how long their effect lasts, and that this ordering follows from how each one works: how long the material stays in the joint, how long it keeps signaling, and how long the patient actually feels better. It is a way of organizing what the literature reports about duration, not a claim that one therapy is more effective than another.

Two points need to be clear from the start. First, these therapies are not equivalent, and we do not treat them as such. HA has decades of regulatory and guideline history; PRP is recommended, with reservations, for selected patients; and cell-based products such as BMAC and SVF still sit largely outside routine guideline endorsement, because of their regulatory status, the complexity of their preparation, and the limited high-quality evidence available. The ESSKA-ORBIT consensus, for instance, places PRP before cell-based therapy in the treatment sequence [12]. Comparing how long their effects last is not the same as saying they are interchangeable.

Second, how long any of these injections works depends heavily on the patient. Osteoarthritis is not one disease. Radiographic grade, the presence of synovitis or effusion, the patient’s metabolic profile, and the type of pain (nociceptive, inflammatory, centrally sensitized, or neuropathic-like) all affect how long relief lasts [20]. We return to these factors in each section, but it is worth stating up front that the ordering we propose describes broad tendencies across mixed populations, not what will happen in an individual patient.

### Scope and Search Strategy

This is a narrative review, so the framework we propose is interpretive rather than the product of a formal systematic search. To keep our sources transparent, we searched PubMed/MEDLINE, Scopus, and the Cochrane Library for English-language work published up to April 2025, and updated key references by hand through June 2025. We combined terms for knee osteoarthritis and intra-articular injection with each therapy (hyaluronic acid, viscosupplementation, platelet-rich plasma, injectable platelet-rich fibrin, bone marrow aspirate concentrate, stromal vascular fraction, and ozone) and with terms for rheology, residence time, growth factor release, and duration of benefit. We gave priority to systematic reviews, randomized trials, and mechanistic studies, and we screened the reference lists of the articles we found. We deliberately left out corticosteroids and simple analgesics: their action is largely immunosuppressive or purely symptomatic and their place in the guidelines is already settled, so they fall outside the biological and physicochemical comparison we set out to make.

## 2. Conceptual Basis for Therapeutic Persistence

The clinical behavior of intra-articular therapy cannot be fully understood by its symptomatic effect alone. Particularly in KOA, the manifestation of clinical outcomes and the duration of benefits relies on more than just simple analgesia. It is also shaped by how the injected product interacts with the synovial microenvironment, how long it remains active within the joint, how it responds to mechanical loading, and whether it merely modulates symptoms or also influences other local biological processes [21,22]. For that reason, the idea of therapeutic persistence is more useful than a rigid comparison of isolated outcomes.

From a practical perspective, therapies may differ in the timing of their effects. Some appear to act quickly but fade earlier, while others may take longer to express their benefit yet maintain it for a more extended period [23]. This difference is not random. It likely reflects the underlying composition of each therapy, the way it is prepared, and the type of signal it delivers to the joint. A fluid with mainly physicochemical action may act differently from a biomaterial that combines mechanical support with biological signaling, and both may behave differently from a product that relies on cellular and trophic activity [24,25].

In this context, HA-based formulations provide a useful example. Conventional HA primarily acts through lubrication, shock absorption, and modulation of the synovial microenvironment [26]. More advanced formulations, however, may also exhibit longer residence time, improved viscoelastic recovery, and greater resistance to enzymatic degradation, which may extend their intra-articular activity and alter the clinical profile of response [27]. These features help explain why not all HA products should be treated as equivalent.

Platelet-based therapies add another layer of complexity. Their activity is not limited to one mechanical effect, especially due to the fact that they can release a broad range of bioactive mediators over time [16]. In similar fashion, cell-rich products may sustain their effect through trophic and paracrine signaling rather than immediate lubrication or short-lived symptom control [28,29]. This suggests that the temporal pattern of benefit may be linked to the biological organization of the therapy itself.

Therefore, when different intra-articular therapies are discussed side by side, the most sensible approach is not to force a universal ranking, but to interpret them according to their expected temporal behavior. A short-term response, an intermediate response, or a more prolonged response may all be plausible depending on the mechanism involved. That is the lens used in the present review.

Because these mechanisms operate on different timescales, it helps to separate the notions of duration that the literature tends to merge under the single word persistence. We distinguish five temporal parameters that are frequently conflated when therapies are compared:

**Physicochemical residence time:** The interval during which the injected material remains physically present within the articular cavity.

**Biological signaling window:** The period over which growth factors, cytokines, or paracrine mediators released by the product remain measurably active.

**Time to peak clinical response:** The interval between injection and maximum symptomatic improvement.

**Duration of clinically meaningful benefit:** The time during which patient-reported outcomes remain above the minimum clinically important difference (MCID).

**Time to reinjection or treatment failure:** The point at which function returns to the pre-injection baseline, or a clinical decision is made to repeat or escalate treatment.

**Figure 1:**
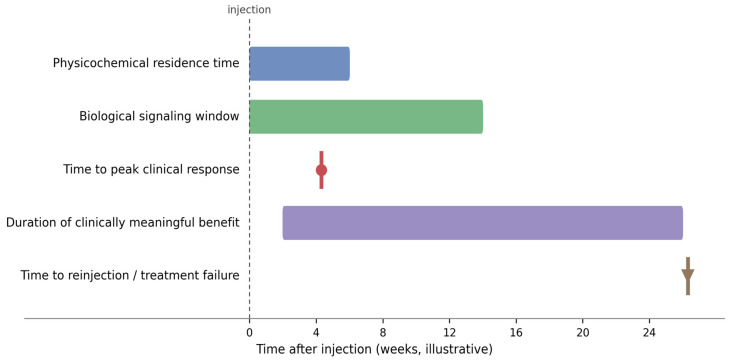
Conceptual relationship between the five temporal parameters used in this review. Within a single injection cycle, physicochemical residence, biological signaling, time to peak response, duration of clinically meaningful benefit, and time to reinjection may overlap or diverge and are not necessarily correlated. Positions are illustrative and not drawn from a single dataset.

These parameters are not always correlated. A product may have a short physical residence yet a longer clinical benefit, or a delayed peak with a sustained effect, and most published studies report only one or two of them. Throughout Section 3, Section 4, Section 5 and Section 6 we indicate which parameter a cited duration actually refers to, rather than treating them as a single output. Figure 1 illustrates how the five parameters may overlap, precede, or diverge from one another within a single injection cycle.

## 3. Ozone Therapy

Ozone arguably occupies the least durable end of the intra-articular therapeutic spectrum discussed here. In KOA, it has been explored as a minimally invasive intervention with proposed anti-inflammatory and analgesic properties, supported by mechanistic evidence involving redox modulation and inflammatory pathway regulation [30,31]. Current evidence characterizes ozone primarily as a biologically active oxidant capable of attenuating oxidative stress and downregulating inflammatory signaling [30,31,32], not a structural biomaterial designed for prolonged intra-articular persistence. Systematic reviews and evidence syntheses have consistently reported clinical benefits, particularly for pain reduction, while also emphasizing the limited methodological quality of many available studies and the uncertainty surrounding direct comparisons with other intra-articular therapies [33,34,35,36].

Within the therapeutic framework proposed here, ozone is best interpreted as a symptom-oriented intervention with predominantly short-duration biological activity [37,38]. Previous studies have demonstrated meaningful improvements in pain and function, with some trials reporting sustained benefits for up to six months [39,40]. However, current evidence does not support the notion of prolonged biological persistence comparable to that associated with more complex orthobiologic strategies. A recent randomized clinical trial demonstrated that different ozone concentrations may produce similar clinical outcomes without clear dose superiority [41]. This observation suggests that increasing ozone concentration does not necessarily translate into greater therapeutic benefit and reinforces the concept that its effects are primarily mediated through early biological signaling and inflammatory modulation rather than prolonged intra-articular residence or sustained structural activity.

Accordingly, ozone can be positioned as the lower anchor of the intra-articular durability continuum proposed in this review. Although it remains a relevant therapeutic option for KOA, its role is more adequately interpreted as a transient, symptom-focused approach and less like an intervention expected to maintain extended intra-articular activity. This distinction becomes particularly important when establishing comparisons with more complex therapies designed to exert longer biological or structural effects.

Ozone is the weakest mechanistic entry in this hierarchy, and its short-end position rests more on the transient nature of its redox activity and its physicochemical half-life in biological fluids than on a consistent duration-of-benefit literature. Three components of its reported effect are worth separating. The first is a direct oxidative and anti-inflammatory action, for which there is plausible preclinical support [30,31,32]. The second is an indirect analgesic effect mediated by modulation of nociceptive signaling, for which the clinical evidence is limited. The third is the placebo and contextual component intrinsic to any intra-articular procedure, which is difficult to isolate for ozone given the scarcity of validated sham comparators. Of the five parameters defined above, the one most relevant to ozone is the biological signaling window, which appears short; claims about the duration of clinically meaningful benefit remain correspondingly uncertain, and for this reason ozone is rated as providing insufficient or speculative evidence in Table 1.

**Modifying factors.** Reported responses to ozone vary with protocol (concentration, volume, number of sessions) and probably with baseline inflammatory status, although the available trials rarely stratify by synovitis, effusion, or pain phenotype, which makes patient-level prediction unreliable.

Taken together, ozone functions as the lower anchor of the continuum: a short-acting, symptom-oriented option that also sets up a contrast with the next class, where physicochemical residence and mechanical function become central rather than incidental.

## 4. Hyaluronic Acid-Based Therapies

HA remains a popular orthobiologic therapy in the arsenal of selected populations of patients with KOA due to its safety profile, ease of application, and potential to alleviate pain and improve joint function [42]. Historically, its clinical rationale has been associated with viscosupplementation, a concept based on the restoration of the viscoelastic properties of synovial fluid [43,44]. However, the progressive evolution of HA formulations has shown that the behavior of these products extends beyond simple lubrication, especially when differences in molecular architecture and rheological properties are considered [45,46].

By default, conventional HA formulations mainly exert mechanical and homeostatic effects within the osteoarthritic joint [46]; through enhanced lubrication and cushioning, HA may decrease frictional load on cartilage and transiently improve joint biomechanics [26]. In addition, it can modulate synovial inflammation, interact with CD44 receptors, and contribute to restoration of joint homeostasis [47]. Nevertheless, the magnitude and persistence of these effects appear to vary substantially among commercially available products, which differ in molecular weight, concentration, source, and degree of crosslinking [46].

This variability has contributed to increasing interest in the physicochemical behavior of HA formulations. Traditional classifications based exclusively on molecular weight may not adequately explain clinical performance, since rheological and tribological properties also influence intra-articular behavior [48]. Bonnevie et al. demonstrated that frictional characterization may correlate more closely with clinical outcomes than viscosity alone, suggesting that lubrication efficiency could represent a relevant determinant of therapeutic response [48]. This observation supports the idea that HA products with similar molecular weight may still behave differently under physiological mechanical stress.

In this context, modified HA hydrogels have emerged as an attempt to improve biomechanical resilience and prolong intra-articular activity. Among these formulations, HYADD4-based hydrogels represent an important example of structural modification designed to alter therapeutic behavior. Unlike conventional linear HA or rigid chemically crosslinked products, HYADD4 contains hydrophobic hexadecyl modifications that promote amphiphilic interactions between polymer chains, generating a dynamic physical hydrogel stabilized by reversible intermolecular associations [49,50].

This architecture appears to influence several relevant properties. Experimental studies have demonstrated improved viscoelastic recovery and resistance to repeated mechanical loading compared with conventional viscosupplementation strategies [49]. Instead of behaving like a static gel, the material may reorganize its internal structure after cyclical stress, a characteristic that has been described as a mobile reticulum system [49]. This feature may be particularly relevant in mechanically demanding joints such as the knee, where repetitive loading continuously challenges intra-articular biomaterials.

Additional studies have suggested that modified HA derivatives may also exhibit biological activity beyond mechanical supplementation. Hyadd C16 derivatives demonstrated inhibitory effects against matrix metalloproteinases (MMP) and hyaluronidases, including MMP13 and hyaluronidase 2 [51]. These enzymes are directly involved in ECM degradation during osteoarthritis progression [51]. Such findings raise the possibility that some modified hydrogels may influence not only symptom control, but also the local catabolic microenvironment within the joint.

Clinical investigations have reported improvements in pain and function following treatment with modified HA hydrogels in patients with KOA, including physically active populations [50]. Imaging studies have also explored potential effects on cartilage metabolism [52] and meniscal tissue organization [53]. Although these preliminary findings should be interpreted cautiously, they still reinforce the concept that different HA formulations may exhibit distinct temporal and biological behaviors.

From a temporal perspective, HA-based therapies appear to occupy an intermediate position between transient symptom modulation and more prolonged biologically active interventions. Conventional formulations often demonstrate clinical improvement over weeks to months [54], whereas modified hydrogels with enhanced rheological recovery and enzymatic resistance may potentially sustain their intra-articular activity for longer periods [49]. Nevertheless, substantial heterogeneity among formulations and study protocols still limits direct comparison across products and prevents definitive conclusions regarding superiority or duration of effect [54].

Two points deserve emphasis so that formulation properties are not read as clinical outcomes. First, rheological superiority measured in the laboratory does not automatically translate into longer clinically meaningful benefit; frictional and viscoelastic advantages describe how a product behaves under load, not how long a patient improves [48]. Whether the rheological advantages of modified hyaluronic acid hydrogels translate into meaningfully longer clinical benefit than conventional formulations remains to be established in adequately powered, independent head-to-head trials. Where possible we have drawn on rheological and clinical studies not affiliated with manufacturers, and we treat the extended persistence attributed to modified hydrogels as a physicochemical hypothesis rather than a demonstrated clinical advantage.

**Patient selection.** The clinical response to hyaluronic acid is not uniform across the osteoarthritic population. Benefit is generally more consistent in early-to-moderate disease (Kellgren–Lawrence I–II) than in advanced joints, and it tends to be attenuated in knees with active synovitis or large effusions, where aspiration or an anti-inflammatory step may be more appropriate before viscosupplementation. Obesity and metabolic syndrome accelerate hyaluronan degradation and may shorten residence time, and mechanical malalignment creates a loading environment that reduces the effective lubrication window [46,47]. These modifiers act mainly on physicochemical residence and on the duration of clinically meaningful benefit, rather than on the intrinsic properties of the product.

On balance, hyaluronic acid occupies the mechanical and physicochemical middle of the continuum, with modified hydrogels offering a plausible but unproven extension of persistence. Platelet-derived products introduce a different logic, one in which the relevant timescale is set by biological signaling rather than by lubrication or residence.

## 5. Platelet-Derived Therapies

Platelet-derived orthobiologics have attracted substantial interest in the field of musculoskeletal medicine because they are able to deliver a concentrated mixture of growth factors, cytokines, and other bioactive proteins directly into the joint [8,16]. Unlike viscosupplementation, which primarily exerts mechanical and rheological effects, platelet-based therapies aim to modulate the biological milieu of the osteoarthritic joint [55].

PRP has been the most extensively studied product within this category. Although preparation protocols remain highly heterogeneous, PRP aims to concentrate platelets and their associated signaling molecules above baseline physiological levels [56,57]. Once activated, platelets release several mediators involved in tissue repair and inflammatory modulation, including platelet-derived growth factor (PDGF), transforming growth factor-beta (TGF-β), vascular endothelial growth factor (VEGF), and insulin-like growth factor-1 (IGF-1) as examples [58]. These mediators are known to influence synovial inflammation, cellular recruitment, ECM turnover, and local anabolic activity within the joint [16].

Regarding treatment longevity, PRP appears to exhibit a clinical profile which differs from conventional intra-articular therapies. Usually, its effects tend not to produce immediate symptomatic relief but to actually emerge progressively over weeks, potentially persisting for longer follow-up periods compared with certain viscosupplementation strategies [55,59]. This delayed yet sustained response pattern supports the concept that platelet-based therapies depend less on immediate biomechanical supplementation and more on gradual biological modulation.

However, interpretation of the literature remains challenging because PRP is not a uniform product [60]. Important variables such as platelet concentration, leukocyte content, activation method, centrifugation protocol, injection number, and interval between applications may significantly alter the biological characteristics of the final preparation [56]. Consequently, studies evaluating PRP often demonstrate inconsistent outcomes and substantial methodological variability, limiting direct comparison between investigations [58,61].

Injectable PRF has emerged as a related strategy with distinct structural characteristics. In contrast to PRP, iPRF contains a fibrin network capable of forming a more organized three-dimensional scaffold after injection [7,8]. This fibrin architecture may prolong growth factor release and potentially increase local biological persistence within the joint microenvironment [7,8]. Studies have suggested that fibrin-based matrices may support cellular migration, tissue interaction, and sustained signaling activity, potentially extending the duration of biological effects compared with liquid platelet preparations [7,8].

The interaction between platelet derivatives and biomaterial science has become increasingly relevant in recent years. Hybrid concepts combining HA and platelet concentrates have attempted to integrate mechanical supplementation with biological signaling, creating functional hydrogels that may simultaneously improve lubrication and modulate tissue repair processes [62,63,64]. This convergence reflects a broader shift within the realm of orthobiologics, where therapies are progressively being interpreted not only as injectable solutions, but also as dynamic biological systems capable of interacting with the ECM and synovial microenvironment.

Clinically, platelet-derived therapies are often associated with intermediate to prolonged temporal response profiles, particularly in patients with mild-to-moderate osteoarthritis. Nevertheless, the persistence of benefit remains difficult to standardize because of heterogeneity in preparation methods and outcome reporting. Current evidence suggests that these therapies may provide more sustained symptomatic improvement than transient anti-inflammatory approaches, although definitive conclusions regarding superiority and exact duration are still premature.

Taken together, platelet derivatives occupy a prominent position between purely mechanical intra-articular therapies and more complex cellular interventions. Their temporal behavior may be determined by progressive biological signaling, matrix interaction, and growth factor release kinetics rather than immediate physicochemical supplementation alone.

Although PRP and iPRF are often discussed together, they are better understood as related but mechanistically distinct products. PRP is a platelet-enriched liquid concentrate whose active fraction is the pool of soluble growth factors released when platelets are activated, so its signaling is comparatively front-loaded. iPRF is a fibrin-based construct that forms a physical matrix after injection and provides a scaffold for more gradual, sustained growth factor release and cell recruitment [7,8]. In terms of the parameters defined earlier, the fibrin architecture of iPRF is expected to lengthen the biological signaling window relative to liquid PRP, although direct head-to-head clinical comparisons remain limited. A longer duration of reported benefit should not be equated with biological superiority; patient selection, osteoarthritis stage, inflammatory phenotype, rehabilitation, and the choice of comparator are powerful confounders in the published trials.

**Preparation variability.** The temporal behavior of platelet products is inseparable from how they are made. Spin speed and the number of centrifugation cycles determine platelet concentration and leukocyte content; the activation method (calcium chloride, thrombin, or the endogenous activation characteristic of iPRF) shapes the kinetics of growth factor release; and fibrin architecture, injection number, and inter-injection interval further modify the effective signaling window [56,58]. Reporting frameworks such as MARSPILL were proposed precisely because these variables, if left undocumented, make the comparison of duration across studies unreliable [56].

**Modifying factors.** Response duration to platelet products is influenced by disease stage, synovial inflammatory status, and pain phenotype, with early to moderate osteoarthritis and an inflammatory rather than a centrally sensitized pain pattern generally associated with more durable improvement [20].

Platelet products therefore sit between the physicochemical and cellular ends of the continuum, with iPRF plausibly extending signaling beyond liquid PRP. The next step along the continuum is toward products whose persistence depends not on a finite pool of mediators but on living cells and their ongoing paracrine output.

## 6. Cellular Orthobiologics

Cell-based products represent biologically complex intra-articular therapies currently available for KOA. Among these approaches, BMAC and SVF have attracted growing interest due to their ability to influence the osteoarthritic joint through trophic, immunomodulatory, and regenerative signaling mechanisms [18,65]. In contrast to therapies primarily dependent on lubrication or short-term biochemical modulation, these products are generally proposed as biologically active systems capable of interacting with the intra-articular microenvironment over longer periods.

### 6.1. Bone Marrow Aspirate Concentrate (BMAC)

BMAC is obtained through concentration of bone marrow-derived cellular components, typically including mesenchymal stromal cells (MSCs), hematopoietic cells, platelets, cytokines, and growth factors [66]. Although MSCs are often highly praised, current evidence suggests that the therapeutic effect of BMAC is likely achieved through paracrine and trophic activity and not direct tissue replacement [67,68]. These signaling pathways may influence synovial inflammation, ECM metabolism, angiogenic balance, and cellular communication within the osteoarthritic joint [66,67,69].

### 6.2. Stromal Vascular Fraction (SVF)

SVF extracted from adipose tissue represents another heterogeneous cellular product containing adipose-derived stromal cells, pericytes, endothelial progenitor cells, immune regulatory cells, and ECM components [70]. Similar to BMAC, its proposed activity extends beyond a simple cell delivery concept. The presence of multiple biologically active cell populations and matrix-associated factors has led to the hypothesis that SVF may function as a supportive biological niche capable of sustaining prolonged intra-articular signaling activity [18,70]. Interest in SVF has also been driven by the relative ease of adipose tissue harvesting and its higher MSC yield compared with bone marrow [18].

In terms of clinical durability, both BMAC and SVF are frequently associated with longer lasting clinical responses in comparison with more transient intra-articular interventions [65,71]. This assumption is largely based on the idea that cellular and paracrine interactions may persist beyond the initial injection period, potentially maintaining biological activity within the joint microenvironment over time [72,73]. In clinical practice, these therapies are often perceived as slower acting but potentially more durable interventions, particularly in selected patients with earlier stages of degeneration.

Nevertheless, interpretation of the current literature requires caution. Considerable heterogeneity exists regarding harvesting techniques, processing methods, cellular concentration, injection protocols, and characterization standards [67,74,75]. The precise cellular composition of the injected product is often not fully characterized, making reproducibility and comparison particularly difficult. In addition, outcome measures and follow-up intervals also vary substantially across publications.

Another important observation involves the frequent use of regenerative terminology that may overstate the strength of current evidence. Although the literature has documented improvements in pain, function, and imaging-related parameters following BMAC or SVF injections, the extent to which these findings reflect true cartilage regeneration or structural disease modification remains controversial. At present, most available data support symptomatic and functional improvement over confirmed restoration of native joint architecture.

Despite these drawbacks, cellular orthobiologics occupy the most biologically complex end of the intra-articular therapeutic spectrum discussed in this review. Their proposed temporal behavior is less dependent on immediate mechanical supplementation and more closely related to sustained trophic signaling, immunomodulation, and interaction with the osteoarthritic microenvironment. For this reason, they are frequently interpreted as therapies with potentially prolonged persistence profiles, although stronger comparative studies remain necessary to clarify the magnitude and durability of their effects.

Grouping BMAC and SVF too closely obscures differences that matter for both biology and regulation. They differ in tissue source, cellular composition, stromal and immune content, and processing, and the regulatory distinction within SVF is particularly consequential: mechanically processed SVF is generally treated as minimally manipulated, whereas enzymatically isolated SVF is classified as a more-than-minimally manipulated cellular product under FDA and EMA frameworks and follows a different, more demanding regulatory pathway [74,75,76]. These distinctions influence what is actually injected and how reproducible it is, and therefore bear directly on any claim about the duration of effect.

**Modifying factors.** For both products, response duration is expected to depend on osteoarthritis grade, inflammatory and synovial status, body-mass index and metabolic phenotype, age, activity level, joint alignment, and meniscal integrity. The relevant temporal parameter here is the biological signaling window sustained by trophic and paracrine activity, which is conceptually distinct from the physicochemical residence that governs viscosupplements.

## 7. Interpreting the Temporal Hierarchy of Intra-Articular Therapies

**Figure 2:**
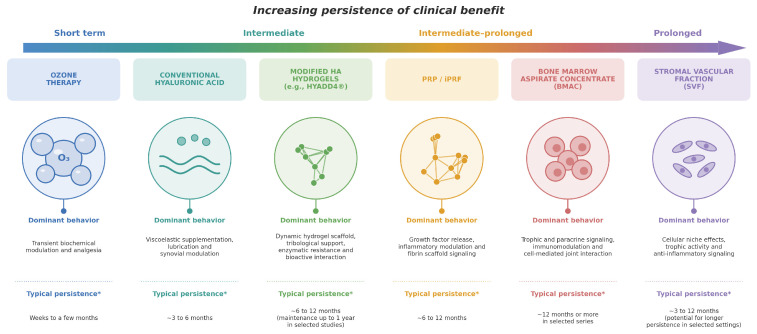
**Proposed temporal hierarchy of intra-articular therapies for knee osteoarthritis.** * Note: Persistence windows represent commonly reported ranges of clinical benefit reported in the available literature.

Based on the currently available literature, intra-articular therapies may be interpreted within a conceptual temporal continuum ranging from transient biochemical modulation to more prolonged biologically active responses. Although substantial heterogeneity exists across studies, recurring patterns related to persistence of clinical benefit and underlying mechanisms can still be identified. These relationships are summarized in Figure 2 and Table 1 immediately below. A horizontal timeline of the proposed hierarchy, with reported ranges of clinical benefit and their uncertainty for each therapeutic class, is provided as Supplementary Figure S1.

The interpretations discussed below are derived from the evidence summarized in Table 1 and the preceding sections.

At the shorter end of this continuum, ozone therapy is typically associated with rapid symptomatic modulation and relatively limited persistence. Its effects are believed to be related primarily to oxidative and anti-inflammatory pathways rather than prolonged intra-articular residence or structural interaction with joint tissues. While some studies have reported clinical improvement extending for several months, the overall therapeutic profile of ozone is generally interpreted as less durable when compared with viscosupplementation and orthobiologic strategies.

**Table 1:** **Comparative mechanistic and temporal characteristics of intra-articular therapies for knee osteoarthritis.** The table has been expanded to separate primary mechanism from temporal driver and to add columns for protocol basis, supporting references, evidence strength (adequate, limited, or insufficient/speculative), and key patient-level modifiers.

Therapy	Primary Mechanism/ Temporal Driver	Commonly Reported Persistence of Clinical Benefit	Protocol Basis	Supporting References	Evidence Strength	Key Patient-Level Modifiers
**Ozone therapy**	Redox modulation and anti-inflammatory signaling; driver: short redox half-life, minimal structural interaction	Short: benefit often within 8 weeks, up to 3–6 months in some reviews	Usually repeated sessions	Lopes de Jesus et al., 2017 [77]; Arias-Vazquez et al., 2019 [38]	Insufficient/speculative	Baseline inflammatory status; protocol; rarely stratified
**Conventional hyaluronic acid (HA)**	Lubrication and viscoelastic supplementation; driver: residence time and rheology	Approximately 3–6 months; trajectory from week 4 to week 24	Single injection or standard 3-injection course	Bannuru et al., 2011 [78]; Costa et al., 2024 [46]	Adequate	KL grade; synovitis/effusion; obesity/metabolic syndrome; malalignment
**Modified HA hydrogels/HYADD4**	Dynamic physical hydrogel, tribological support, enzymatic resistance; driver: self-repairing amphiphilic network	Approximately 6–12 months in selected studies, up to 1 year	Single injection or short course	Benazzo et al., 2016 [79]; Bernetti et al., 2021 [80]	Limited	Loading environment; same patient modifiers as conventional HA
**Platelet-rich plasma/injectable platelet-rich fibrin (PRP/iPRF)**	Growth factor release (PRP) and fibrin-scaffold-mediated sustained release (iPRF); driver: signaling kinetics	Commonly 6–12 months; some comparative data to 24 months	Typically 1–3 injections	Dai et al., 2017 [81]; Wang & Yao, 2025 [59]; Verra et al., 2018 [82]; Cheeva-akrapan & Turajane, 2023 [83]	Limited to adequate (PRP); limited (iPRF)	Preparation variables; KOA stage; inflammatory/pain phenotype
**Bone marrow aspirate concentrate (BMAC)**	Trophic and paracrine signaling, immunomodulation; driver: sustained cell-mediated signaling	Around 12 months; randomized data to 24 months	Usually single injection	Shapiro et al., 2017 [84]; Han et al., 2024 [85]	Limited	Cell composition; processing; disease stage; metabolic phenotype
**Stromal vascular fraction (SVF)**	Cellular niche and trophic/anti-inflammatory signaling; driver: sustained paracrine activity	Approximately 3–12 months; benefit at 12 months in retrospective data	Usually single injection; enzymatic vs. mechanical processing differs	Boada-Pladellorens et al., 2022 [86]; Jeyaraman et al., 2024 [87]; Lu et al., 2025 [88]	Limited	Isolation method/regulatory class; viability; host factors

Note: Persistence windows are presented as commonly reported ranges of clinical benefit instead of fixed or universally reproducible durations because of heterogeneity in study design, patient populations, formulations, and outcome measures.

Conventional HA formulations appear to occupy an intermediate temporal position. Their clinical activity is mainly associated with lubrication, viscoelastic supplementation, and restoration of synovial homeostasis. In most studies, symptomatic improvement is commonly reported over periods ranging from several weeks to approximately 6 months, although variability among formulations remains considerable. Molecular weight, concentration, crosslinking characteristics, and rheological behavior may all influence intra-articular persistence and clinical response.

Modified HA hydrogels may represent a distinct category within viscosupplementation because of their more complex structural organization. Amphiphilic derivatives stabilized by dynamic hydrophobic interactions have demonstrated improved viscoelastic recovery, enhanced tribological behavior, and greater resistance to enzymatic degradation in experimental models. These characteristics may contribute to longer persistence of intra-articular activity and potentially more sustained clinical responses compared with conventional HA products. Nevertheless, current evidence remains heterogeneous, and definitive superiority among formulations cannot yet be established.

Platelet-based orthobiologics such as PRP and iPRF appear to exhibit a temporal profile that differs from therapies based predominantly on mechanical supplementation. Their activity depends on progressive biological signaling mediated by growth factor release, inflammatory modulation, and interaction with the ECM. Clinical improvement is frequently reported over intermediate to long term follow-up periods, commonly extending from 6 to 12 months in selected studies, although important variability persists because of differences in preparation protocols and patient characteristics. The fibrin architecture associated with iPRF may further contribute to prolonged local biological activity through sustained release kinetics and scaffold formation.

At the more prolonged end of the proposed continuum, BMAC and SVF are frequently described as therapies capable of maintaining longer lasting clinical responses. This interpretation is largely based on their trophic and paracrine activity, cellular heterogeneity, and potential interaction with the osteoarthritic microenvironment. Some studies have reported sustained symptomatic improvement extending beyond 12 months following cellular orthobiologic injections, particularly in patients with less advanced disease stages. However, methodological heterogeneity remains substantial, and current evidence still does not support definitive conclusions regarding structural regeneration or universal long-term superiority.

Importantly, the temporal patterns discussed in this review are not proposed as rigid or universally reproducible durations. Differences in osteoarthritis severity, patient related factors, rehabilitation protocols, injection techniques, product preparation methods, and outcome measures may significantly alter clinical response. In addition, many studies evaluate repeated injection cycles instead of isolated interventions, further complicating direct comparison among therapies.

Despite these limitations, a mechanism-driven interpretation of temporal behavior may still provide clinically useful insight. Intra-articular therapies should not be viewed as interchangeable interventions because current evidence suggests that they may represent biologically and mechanically distinct strategies with different persistence profiles. Understanding these differences may contribute to more individualized treatment selection and may also guide future development of orthobiologic and biomaterial-based therapies for knee osteoarthritis.

## 8. Clinical Implications and Future Perspectives

The proposed temporal hierarchy should be viewed as a pragmatic framework for interpreting current evidence and informing clinical decision-making, not as a fixed ranking of efficacy. In KOA, the expected persistence of benefit may help align the choice of therapy with the clinical goal, whether that goal is early symptom control, intermediate support, or a more durable biological response. This perspective is especially relevant in a field where products are often grouped under the same therapeutic label despite having very different structural and biological properties.

It is useful to place this framework alongside current guidelines. The 2019 ACR/Arthritis Foundation and 2019 OARSI recommendations conditionally support conventional hyaluronic acid and, in the ACR document, express uncertainty about intra-articular options that lack consistent high-quality evidence, while neither endorses cell-based therapy for routine use [89,90]. The ESSKA-ORBIT consensus is more granular and directly relevant here, since its positioning of PRP ahead of cell-based therapy on grounds of preparation complexity and regulatory status mirrors the ordering that a mechanistic reading of persistence would predict [12]. The temporal hierarchy proposed in this review is not intended to replace guideline-based decision-making but to complement it, by offering a mechanistic rationale for therapy sequencing and for the timing of reinjection.

### 8.1. Clinical Implications and Patient Selection

Translating this framework into practice requires attention to the patient as much as to the product. Radiographic grade matters: earlier disease (Kellgren–Lawrence I–II) tends to respond more durably across classes, whereas advanced degeneration narrows the expected benefit of every intra-articular option. Inflammatory phenotype is a second modifier, as effusive, synovitic knees may respond differently from dry knees, a distinction that is particularly relevant for platelet and cellular products. Safety should be weighed alongside expected persistence: post-injection flare is the most common event with hyaluronic acid and PRP, while marrow or adipose harvesting for BMAC and SVF adds procedural and infection-related risk. Active joint infection contraindicates all of these therapies, and coagulopathy or ongoing anticoagulation warrants particular caution with marrow- and adipose-derived procedures. Decisions about reinjection are best anchored to the temporal parameters defined in Section 2, using time to reinjection or treatment failure rather than a fixed calendar interval.

### 8.2. Future Perspectives

The main challenge for the field is not simply the lack of more studies, but the lack of studies designed to test temporal behavior in a standardized way. Future investigations should prespecify critical time-related checkpoints such as onset of action, peak response, duration of clinically meaningful improvement, and time to reinjection or treatment failure. These measures should be reported alongside baseline osteoarthritis grade, patient phenotype, symptom duration, prior treatment exposure, and follow-up intervals. Without this level of structure, persistence remains a loosely used concept when it should be a testable endpoint.

Framed more precisely, the gap is not a shortage of trials but the near-absence of studies that treat duration of effect as a prespecified primary or co-primary endpoint, with standardized product preparation, injection timing, and outcome measurement at defined temporal checkpoints. The lack of validated temporal endpoints in current outcome frameworks is a concrete illustration of this problem.

A related limitation is that osteoarthritic pain is not a single construct. Nociceptive, inflammatory, centrally sensitized, and neuropathic-like components coexist in varying proportions, and their prevalence in knee osteoarthritis is substantial [20]. A temporal framework that ignores pain phenotype risks is clinically misleading, because a flare-directed intervention and a lubrication- or microenvironment-directed one should not be held to the same expectation of duration. Future trials should incorporate validated pain-phenotype classification as a stratification variable and report outcomes by subgroup.

Product characterization should also become a mandatory part of study reporting. For HA, authors should specify molecular weight, concentration, source, crosslinking status, and relevant rheological or tribological features. For PRP, platelet concentration, leukocyte content, activation method, final volume, and number of injections should be reported in full. For BMAC and SVF, harvest site, processing technique, cell yield, viability, and degree of manipulation should be clearly defined. These variables are not technical extras; they are likely determinants of temporal behavior and may explain why apparently similar therapies behave differently in practice.

A practical way forward is a two-component reporting standard for future temporal studies. The first component is a product descriptor recording the minimum preparation and composition parameters for each class: molecular weight, degree of cross-linking, concentration, and volume for hyaluronic acid; platelet and leukocyte concentration, activation method, volume, and injection number for PRP; and aspiration or harvest site, processing protocol, total nucleated cell count, and viability for BMAC and SVF. The second is a patient descriptor recording the baseline variables most likely to modify duration: Kellgren–Lawrence grade, synovial and effusion status, baseline WOMAC or KOOS, pain-phenotype classification, body-mass index, and prior injections. Alongside this, it is worth adopting the distinction between flare-directed, lubrication-directed, and microenvironment-modifying interventions, since these carry inherently different temporal expectations and should be matched to appropriate comparators and endpoints.

Regulatory context should also be integrated into future work, especially for cell-rich products. Definitions related to minimal manipulation, homologous use, processing limits, and manufacturing standards differ across jurisdictions and may influence what is actually being injected, how reproducible the product is, and how readily the data can be translated into clinical practice [76]. Clear reporting of regulatory category and processing pathway, including the relevant framework used by agencies, such as the United States Food and Drug Administration and ANVISA, would make comparative interpretation more reliable and would help distinguish biologically meaningful products from merely nominally similar preparations.

A more advanced research agenda should move beyond broad class comparisons and test mechanism-based hypotheses. For example, modified HA hydrogels could be compared with conventional formulations using shared follow-up windows and matched clinical endpoints to determine whether rheological design truly alters persistence. Platelet-based therapies should be studied with attention to release kinetics and scaffold behavior, while cellular orthobiologics should be evaluated with explicit links between product composition and temporal response. Imaging and biomarker endpoints can provide important complementary insight to clinical outcomes [91,92], particularly when the objective is to differentiate short-term symptomatic improvement from sustained biological activity.

In this sense, the real contribution of the present review is not a definitive verdict on which therapy lasts the longest, but a more structured way to think about why some therapies appear to persist longer than others. A future literature built around standardized reporting, clearer product definition, and temporally meaningful endpoints would allow this proposed hierarchy to be refined, challenged, or confirmed with much greater confidence.

## 9. Conclusions

Intra-articular therapies for knee osteoarthritis should not be viewed as temporally equivalent interventions. Their clinical persistence appears to reflect differences in physicochemical design, biological signaling, and interaction with the joint microenvironment. Within this framework, ozone occupies the shortest end of the proposed continuum, conventional hyaluronic acid remains an intermediate option, modified hyaluronic acid hydrogels may extend temporal activity through improved structural behavior, and platelet-based or cellular orthobiologics may support more prolonged responses in selected settings.

The available literature does not yet justify a rigid universal ranking. However, it does support the concept that the duration of benefit is influenced by product composition, processing, and mechanism of action. For this reason, the proposed temporal hierarchy should be interpreted as a literature-informed framework that may help organize current knowledge and guide future study design.

A more robust field will depend on standardized reporting of product characteristics, uniform time-related endpoints, and clearer regulatory description of each preparation. Until then, the value of this review lies in providing a structured way to interpret why some therapies appear to act briefly while others seem to maintain benefit for longer periods. That distinction may help clinicians align treatment choice with therapeutic goals and may also support the development of more predictable, mechanism-driven intra-articular therapies.

## Data Availability

No new data generated.

## Supplementary Materials

The following supporting information can be downloaded at https://www.mdpi.com/article/10.3390/gels12070608/s1, Figure S1: Proposed temporal hierarchy of intra-articular therapies for knee osteoarthritis presented as a horizontal timeline with reported ranges of clinical benefit and their uncertainty for each therapeutic class.
