# Supplementary material for: Temporal Hierarchy of Hydrogels and Orthobiologic Therapies for Knee Osteoarthritis
Source: Gels. 2026 Jul 8;12(7):608. doi: 10.3390/gels12070608 (PMC13408274; doi:10.3390/gels12070608)
Supplement: Supplementary file 1 [file gels-12-00608-s001.zip › gels-4396316-supplementary.pdf]

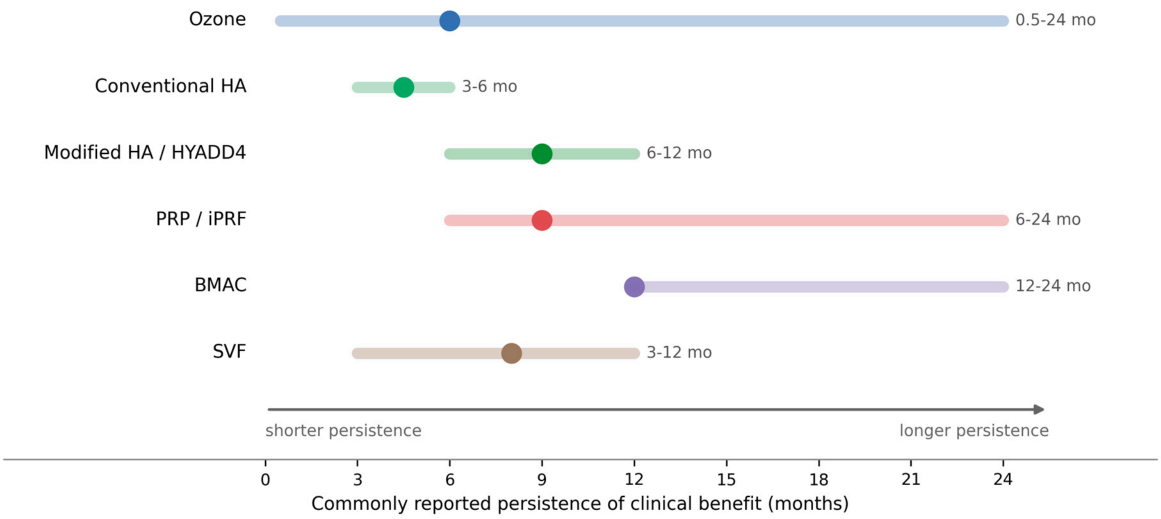

Figure S1. Proposed temporal hierarchy of intra-articular therapies for knee osteoarthritis presented as a horizontal timeline with reported ranges of clinical benefit and their uncertainty for each therapeutic class
